# Current Status and Prospects of Research on Ischemia-Reperfusion Injury and Ferroptosis

**DOI:** 10.3389/fonc.2022.920707

**Published:** 2022-08-25

**Authors:** Lin Hou, Xiaodong Li, Chang Su, Kailin Chen, Maoxing Qu

**Affiliations:** ^1^ Institute of Cancer Stem Cell, Dalian Medical University, Dalian, China; ^2^ First Clinical College, The First Afiliated Hospital of Dalian Medical University, Dalian, China; ^3^ Second Clinical College, The Second Afiliated Hospital of Dalian Medical University, Dalian, China; ^4^ Department of Critical Care Medicine, The Second Afiliated Hospital of Dalian Medical University, Dalian, China

**Keywords:** ischemia-reperfusion injury, Ferroptosis, treatment, diagnosis, mechanisms

## Abstract

The pathogenesis of ischemia-reperfusion injury is not fully understood, most of the current clinical treatment methods mainly relieve symptoms, and cannot prevent fundamentally. The mechanism of Ferroptosis has been extensively studied in recent years, but primarily focused on its therapeutic effects on tumors. After careful comparison, it is easy to find that the symptoms of ischemia-reperfusion injury often accompany by increased lipid peroxidation and increased intracellular iron level are the same as the manifestations of iron-dependent non-apoptotic Ferroptosis. Based on this “coincidence”, we launched this survey. After reading a lot of literature, we found that Ferroptosis is the first step of ischemia-reperfusion injury, and cell necrosis and inflammation are the subsequent steps secondary to Ferroptosis. In this review, we have collected and sorted out the current knowledge about the role and targets of Ferroptosis in the process of ischemia-reperfusion injury. And future studies may be biased towards exploring the use of ferroptosis inhibitors in combination with other treatment options.

## 1 Definition Of Ischemia-Reperfusion Injury

Ischemia Reperfusion Injury (IR) contains two parts: ischemia and reperfusion, that is, after blood flow and oxygen are reconstructed to the anemic area, the ischemic tissue deteriorates excessively and triggers a destructive inflammatory response (1). It is often secondary to myocardial infarction, thrombolytic surgery, organ transplantation, ischemic stroke and other activities, causing damage to the heart, brain, liver, kidney, limbs, small intestine and other parts. In the process of ischemia, to protect essential organs, the body often reduces the blood supply to the skin and mucous membranes. Therefore, ischemia-reperfusion injury of the small intestine and peripheral extremities is more common (2). Although people discovered the existence of reperfusion injury after ischemia in canines as early as 1960, the mechanism of its occurrence is still not fully understood.

## 2 The Mechanism Of Ischemia-Reperfusion Injury

### 2.1 Mechanism of Excessive Free Radical Generation

#### 2.1.1 Succinic Acid Accumulation

Ischemia causes the average tricarboxylic acid circulation in the body to be blocked which leads to the accumulation of succinic acid, the intermediate product. This phenomenon has been confirmed in the myocardium, liver, brain, kidney and other organs (3). With the restoration of oxygen supply during reperfusion, the accumulated succinate in the mitochondria will be rapidly oxidized under the action of succinate dehydrogenase SDH, prompting the respiratory chain complex 1 on the mitochondrial membrane to transport electrons in reverse and generate a large amount of ROS (4).

#### 2.1.2 Neutrophil Aggregation and Activation

Neutrophils in the human body will accumulate in a large number of tissues that have undergone avascular necrosis, and will be activated in large numbers during reperfusion. The oxygen taken by it can generate free radicals under the action of NADPH oxidase and NADH oxidase in the cell, which resulting in further tissue damage. During ischemia, the aerobic metabolic disorder causes a decrease in the production of ATP, and the tissue is obviously acidified, which provides a suitable acidic environment for the Fenton reaction, which is further conducive to the generation of free radicals (5).

#### 2.1.3 Increase in Xanthine Oxidase

Xanthine oxidase (xanthine oxidase, XO) and its predecessor Xanthine dehydrogenase (xanthine dehydrogenase, XD) mainly in the capillary endothelial cells. Under normal circumstances, it mainly exists in the form of xanthine dehydrogenase. During ischemia, a large amount of Ca2+ enters the cell to activate the Ca2+-dependent proteolytic enzyme, which converts xanthine dehydrogenase into xanthine oxidase in a large amount. In the mean time, due to the degradation of ATP, a large amount of hypoxanthine accumulates in the ischemic tissue. When the ischemic tissue restores the oxygen and blood supply, the two can undergo a catalytic oxidation reaction to produce a large amount of oxygen free radicals or reactive oxygen species such as hydrogen peroxide (6, 7).

#### 2.1.4 Eliminate the Negation of the Mechanism

When exposed to natural physiological conditions, the body has a variety of defense measures against free radical damage, such as low molecular free radical scavengers (vitamin E, ascorbic acid), enzymatic scavengers (superoxide dismutase, SOD). The reducing glutathione and reducing coenzyme (NADPH) in the cytoplasm are also used to reduce and eliminate certain free radicals under the action of catalase and glutathione peroxidase. However, those as mentioned above various endogenous active oxygen scavenging mechanisms and their scavenging capabilities have been denied or weakened after the occurrence of ischemia-reperfusion injury (8).

**Figure 1:**
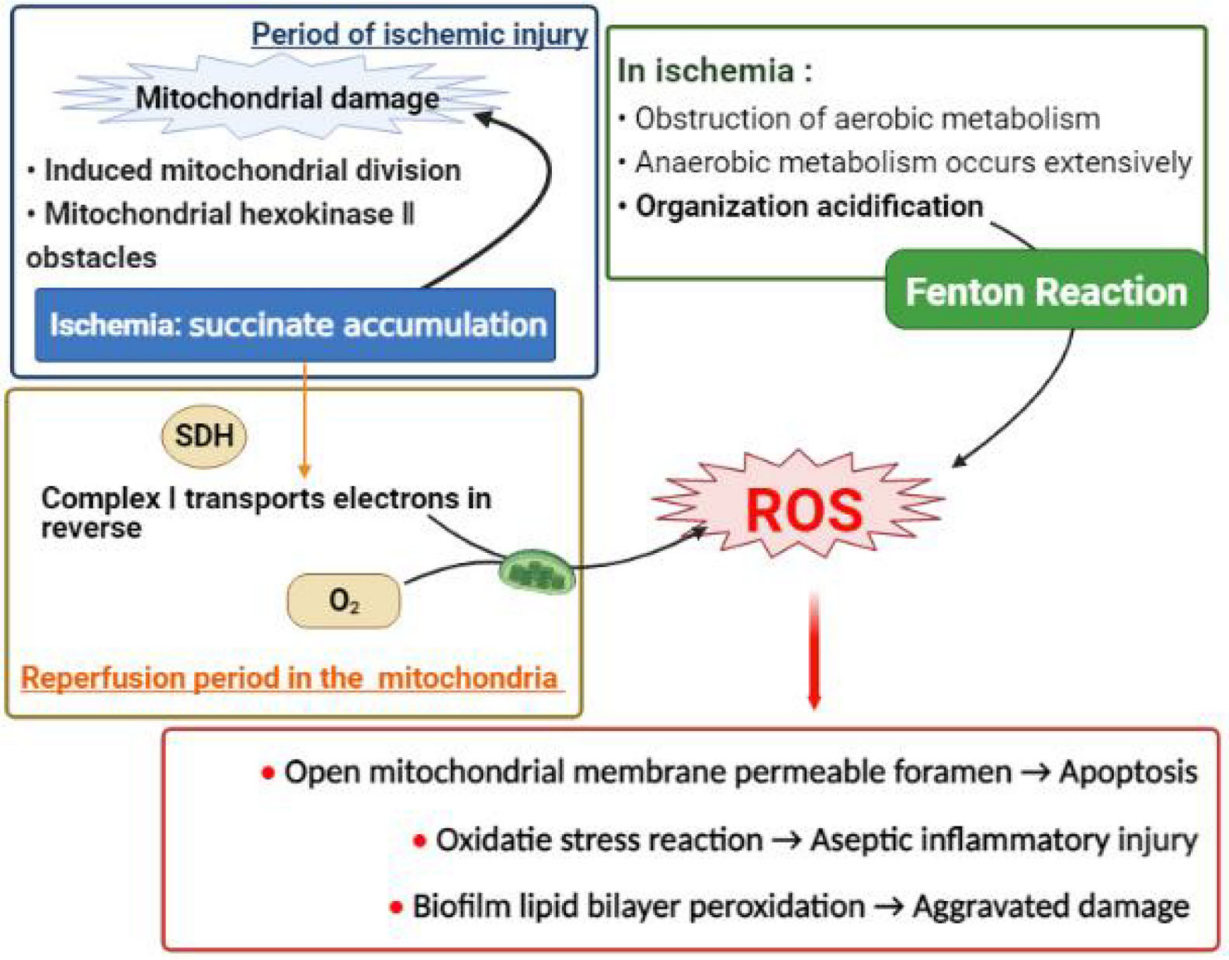
The mechanism diagram of excessive free radical generation (partial). Excessive ROS is a major cause of ischemia reperfusion injury. It can destroy mitochondrial structure and induce cell apoptosis, or participate in oxidative stress response and cause inflammatory damage; and also damage cell morphology and stabilize energy supply by peroxidation of cell membrane. **(A)** A large amount of succinic acid accumulated during ischemia can not only cause initial mitochondrial damage, but also be rapidly oxidized by succinic acid dehydrogenase after the reperfusion oxygen supply is restored, inducing the respiratory chain complex 1 on the mitochondrial membrane to reverse phase transport electrons and produce a large number of ROS. **(B)** At the same time, anaerobic metabolism occurs in most tissues in the ischemic stage, providing favorable acidic conditions for Fenton reaction to produce ROS.

In summary, during the period of reperfusion, the burst of ROS is caused by the joint action of various mechanisms (1). Excessive ROS can cause the opening of the mitochondrial membrane permeable pores and induce cell apoptosis (9), or participate in oxidative stress and cause aseptic inflammatory damage (10); cause lipid peroxidation in the lipid bilayer of the biomembrane. The reaction makes the cell shape and energy supply unable to maintain, further worsening the damage. In addition, excessive ROS can also cause severe adverse reactions such as cross-linking of certain important proteins, chromosomal aberrations, nucleic acid base changes and even DNA breaks (11). See **Figure 1**.

### 2.2 Calcium Overload Mechanism

During the period of ischemia and hypoxia, the body’s energy supply is mainly based on anaerobic metabolism, resulting in significant tissue acidification, and the pH value is observably reduced. Once the blood supply is restored, the pH gradient formed inside and outside the cell promotes the activation of Na+-H+ and Na+-HCO3-transporters, accelerating the efflux of hydrogen ions and the influx of sodium ions, thereby quickly correcting the pH value. However, at this time, the increase of sodium ion concentration in the cell induces Na+-Ca2+ exchange, causing a large number of extracellular calcium ions to the influx and causing calcium overload. Similarly, the large amount of oxygen free radicals produced during the reperfusion period can cause lipid peroxidation of the cell membrane, increase membrane permeability, and promote a large influx of extracellular calcium ions along with the gradient concentration (12).

In addition, mitochondria suffer different degrees of damage during the cell ischemia phase or the reperfusion phase. During ischemia, the accumulation of succinate caused by the disorder of the tricarboxylic acid cycle can cause mitochondrial function damage by inducing mitochondrial division and mitochondrial hexokinase II damage (13). During the period of reperfusion, excess ROS can enhance lipid peroxidation, thus influencing the fluidity of mitochondrial membranes, which leads to mitochondrial dysfunction and loss of energy reserves in cells. The damage of mitochondria makes the energy-dependent plasma membrane and sarcoplasmic reticulum calcium pump run impaired, and excessive Ca2+ in the sarcoplasm cannot be pumped out or absorbed by the sarcoplasmic reticulum, which leads to increased intracellular Ca2+ concentration, causing Ca2+ overload in cells. Further aggravating the damage (7). Calcium overload can activate this dependent proteolytic enzyme and phosphorylase, destroy the structure of the biomembrane, and cause cell apoptosis or necrosis (14).

### 2.3 Other Mechanisms

The body can also activate the immune system by expressing cytokines, adhesion molecules, chemokines and other substances, such as Toll-like receptor activation and the immune response mediated by the complement cascade,resulting in excessive activation of the inflammatory response, resulting in secondary ischemia-reperfusion injury (15). In addition, recent studies have found and confirmed that ischemia-reperfusion injury is often accompanied by an increase in intracellular iron levels, which is related to Ferroptosis in the mechanism of programmed cell death. In addition, reperfusion injury in different parts has its own unique mechanisms, such as brain excitatory amino acid toxicity (1).

### 2.4 Diagnosis of Ischemia-Reperfusion Injury

At present, there are no clinically specific diagnostic criteria for ischemia-reperfusion injury. Moreover, in conjunction with the analysis of clinical cases, it is found that the risk of ischemia-reperfusion injury in the intestines and limbs in severe patients is higher than that of the heart or other organs, and the degree of injury is even more serious. Therefore, we speculate that future clinical diagnostic criteria for ischemia-reperfusion injury may need to be formulated based on the characteristics of different tissues and organs. Take the literature report as an example, and Ferroptosis occurs 30 minutes after reperfusion, as a sign of early reperfusion injury. Perhaps Ferroptosis can be used as a diagnostic basis for intestinal ischemia-reperfusion injury (16).

### 2.5 Prevention and Treatment of Ischemia-Reperfusion Injury

As shown in **Table 1**, most of the existing treatments are symptomatic treatments, which can only alleviate the reperfusion injury caused by a single pathway, but cannot be fundamentally alleviated, and they have many adverse reactions (17).

**Table 1:** Treatment ideas and specific measures for ischemia-reperfusion injury.

Countermeasure ideas	Concrete measures
Restore blood flow as soon as possible and control reperfusion conditions	Try to restore blood flow before reperfusion injury occurs Low pressure, low temperature and low flow rate perfusion with low sodium and high potassium solution
Eliminate or reduce free radicals,	supplement antioxidant substances: coenzyme Q, vitamin E, etc
Reduce calcium overload	using calcium channel blockers
Use cytoprotective agents to enhance tolerance	supplement hexose phosphate and exogenous ATP
Use inhibitors of provocative damage to protect cells	CyclosporinA, abciximab-glycoprotein IIb/IIIa inhibitors
Activate the endogenous protective mechanism	Ischemic preconditioning, post-ischemic preconditioning, remote ischemic preconditioning

## 3 Ferroptosis

### 3.1 The Discovery of Ferroptosis

Ferroptosis is a new type of programmed death mechanism discovered in recent years other than apoptosis and other death pathways. It is usually caused by the accumulation of iron-dependent lipid peroxides to a fatal level (1), iron chelate. The mixture can inhibit this process, so it is called Ferroptosis (18). In 2003, it was first discovered that Erastin has certain cytotoxicity and is related to glutathione metabolism (19). Moreover it was discovered in 2008 that iron chelating agents could inhibit this mode of death (20). Dixon et al. formally named this death mechanism as Ferroptosis based on the above characteristics in 2012 (21). Since 2015, people’s cognition of Ferroptosis has entered a stage of rapid development, and has successively explored the regulation of iron levels in the body, the glutamine metabolism pathway (Xc system), and the influence of lipid metabolism on Ferroptosis (22), And summarized the positive regulators based on NADPH oxidase, p53-p21 axis and the reverse regulators represented by glutathione peroxidase 4, heat shock protein-1 and nuclear factor erythrocyte 2 (23). In 2019, Bersuker, K, Doll, S and others proposed an independent parallel system: the FSP1-COQ10-NAD(P)H pathway, which can work with GPX4 and glutathione to inhibit phospholipid peroxidation and participate in the formation of Ferroptosis (22).

### 3.2 The Characteristics of Ferroptosis

On the morphological level, Ferroptosis is manifested as mitochondria smaller than normal, mitochondrial membrane density is dense, mitochondrial cristae decreases or disappear, and mitochondrial outer membrane ruptures (20). In terms of pathophysiology, it exhibits unique glutathione peroxidase 4 inactivation and lipid peroxide accumulation, as distinct from other forms of cell death (24). No specific markers of Ferroptosis have been found, such as apoptosis (caspase activation) and autophagy (autophagolysosome formation) (22). (See **Table 2** for details).

**Table 2:** Comparison of differences among Ferroptosis, apoptosis, necroptosis, and autophagy.

	Ferroptosis	Apoptosis	Necroptosis	Autophagy
morphology	No cell membrane rupture Cell aggregation Mitochondria shrink lesions; The size of the nucleus is normal; No chromosome condensation	Blistering of cell plasma membrane; Cell aggregation Decreased cell volume and nuclear volume; Nuclear fragmentation, chromosome condensation; Formation of apoptotic bodies (25)	Rupture of cell plasma membrane; Swelling of cytoplasm; Slight chromatin condensation; (25)	No obvious changes in cell plasma membrane; Accumulation of autophagic vacuoles; Chromatin has not been condensed; Autolyzed body formation; (25)
Biochemistry	Accumulation of iron and active oxygen; Activate MAPKs (25); Xc system is suppressed; Reduce the absorption of cystine; Glutathione depletion; Release of arachidonic acid mediator (25);	Activation of Caspase PS Exposure of Oligonucleotide DNA Fragments (25)	The decrease of ATP release level can inhibit the activation of PARP1 (25)	LC3-I to LC3-II conversion substrate degradation (25)
Immunology	Induces an inflammatory response (23)	Anti-inflammatory and immune silence (23)	Mostly to promote the occurrence of inflammation (23)	Mostly anti-inflammatory (23)

### 3.3 The Mechanism of Ferroptosis

**Figure 2:**
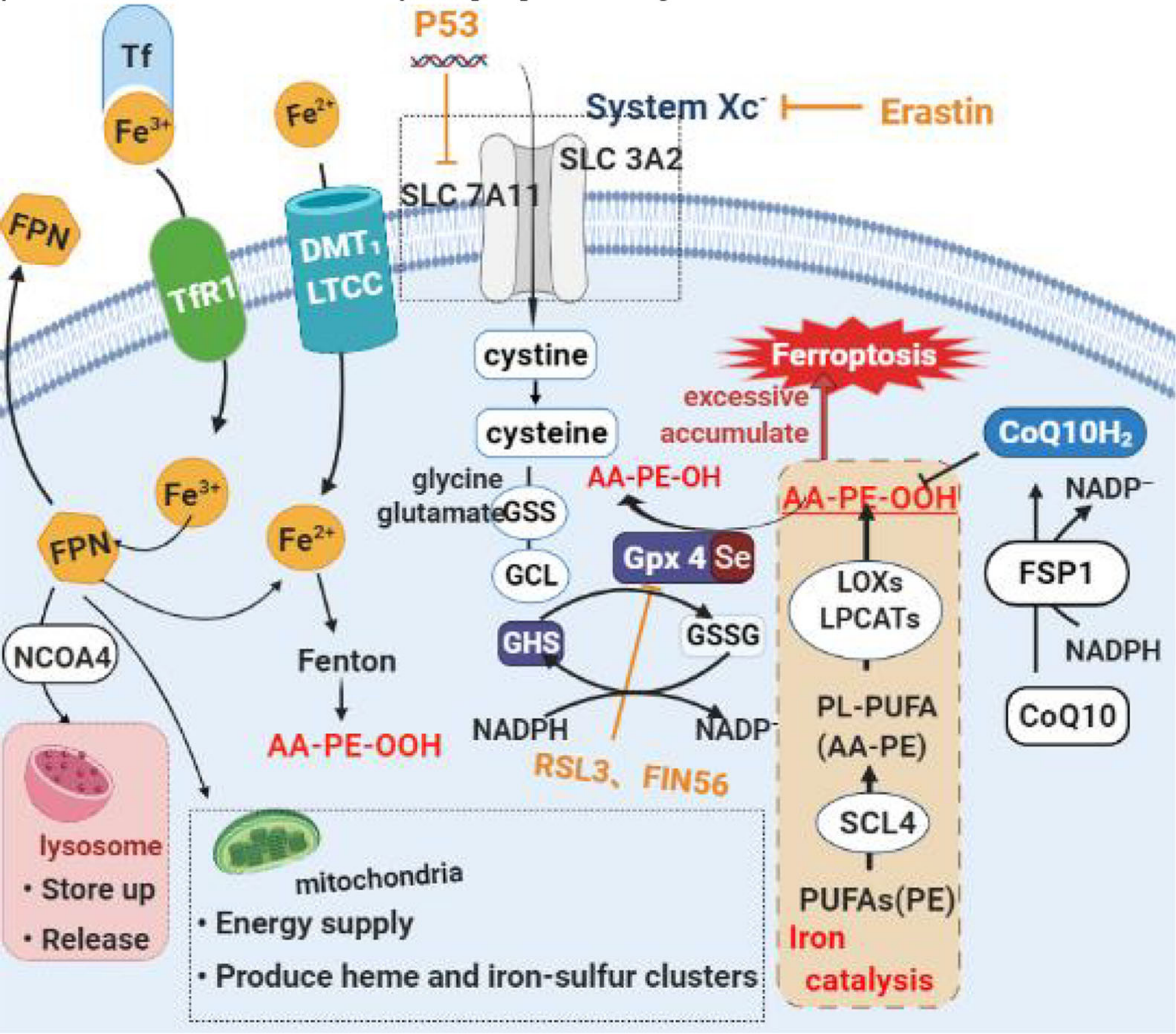
Diagram of the main mechanism of Ferroptosis. **(A)** Lipid peroxide restoration mechanism mainly based on Erastin/Glutathione/Gpx4 axis. Erastin, an iron death inducer, inhibits cystine uptake and subsequent glutathione biosynthesis by inhibiting System Xc-. This reduces the activity of GPX4, accumulates a large amount of lipid oxides, and eventually causes ferroptosis. **(B)** Intracellular free long-chain polyunsaturated fatty acids can undergo acylation lipidation oxidation under the sequential action of ACSL4, LOXs and LPCAT3 to generate lipid peroxidation. Ferroptosis occurs when its level exceeds a threshold. At the same time, iron ions promote the process. **(C)** Normally most of the iron in the matrix is taken up by mitochondria to synthesize heme and iron-sulfur clusters, and a few are reserved in FPN. Ferroptosis is often accompanied by increased transferrin content. This makes a large amount of FPN binded with NCOA4 be transported to lysosome to degrade and release iron, which ultimately leads to the destruction of iron homeostasis, the increase of local iron content and the occurrence of ferroptosis.

The accumulation of iron and lipid peroxides is the main feature of Ferroptosis. It can be understood that the excessive accumulation of peroxides and reaching a certain extent leads to cell death. This process is dependent on the catalysis of iron. Therefore, the mechanism of Ferroptosis can be divided into three parts: lipid peroxide reduction inhibition, lipid peroxide synthesis promotion, and iron catalysis (22). See **Figure 2**.

#### 3.3.1 Mechanisms of Inhibition of Lipid Peroxide Reduction

Glutathione Peroxidase 4 (GPx4) is an endogenous antioxidant that depends on selenium and glutathione. In a physiological state, it can inhibit the occurrence of Ferroptosis by reducing lipid peroxides to corresponding fatty alcohols and removing lipid reactive oxygen species in cells. At the same time, selenium is a necessary substance to maintain its activity, and the lack of trace element selenium in the body will also cause the inactivation of GPx4, and then be sensitive to Ferroptosis (26). In summary, the lipid peroxide reduction inhibition process can also be roughly understood as the inactivation process of GPx4. We will illustrate the specific steps of the process through the effects of several common Ferroptosis inducers, the most representative of which is the Erastin/Glutathione/Gpx4 axis (24).

Erastin mainly exerts an inducing effect by inhibiting the Xc system. This is a cystine/glutamate antiporter composed of XCT (SLC7A11) and 4F2 (SLC3A2), which can excrete glutamate from the cell and transfer cystine into the cell and quickly convert it to cysteine. Acid. Glutamate, cysteine and glycine in the cell are catalyzed by cytoplasmic enzymes, glutamate-cysteine ligase (GCL) and glutathione synthase (GSS) to produce glutathione (26). Therefore, inhibiting this process will lead to a decrease in the synthesis of glutathione using cysteine as a substrate, and glutathione is depleted (25), the activity of GPx4 is fall off, a large number of lipid oxides accumulate, and Ferroptosis. With people’s in-depth understanding of cell metabolism, it has been discovered that the cysteine of some cells can also depend on the production of transsulfuration reactions (1). Therefore, the contradiction that is simply inhibiting the Xc pathway of such cells cannot theoretically induce Ferroptosis provides a direction for scientists’ research. In recent days, studies have found that over and above inhibiting the Xc system, Erastin can also cause Ferroptosis by inhibiting the mitochondrial voltage-dependent anion channel (VDAC). In addition, RSL3 and FIN56, which also act in this pathway, act by combining with GPx4 to inactivate or promote the degradation and inactivation of GPx4, leading to Ferroptosis (18). At the genetic level, P53 inhibits cystine uptake by cells. This process is achieved by down-regulating the expression of SLC7A11 in XC system, resulting in a decrease in glutathione peroxidase activity and enhancing the sensitivity of cells to Ferroptosis. In addition, down-regulation of NrF2 can inhibit the downstream antioxidant protein gene (such as GPx4) encoding transcription (27), thus inducing Ferroptosis.

Similarly, Ferroptosis does not result from inhibition of GPx4 activity alone. Recent evidence indicates that it is necessary to inhibit the Ferroptosis inhibitor protein 1 (FSP1)-coenzyme Q10 (CoQ10)-nicotinamide adenine dinucleotide phosphate (NAD(P)H) pathway and the GPx4/glutathione pathway at the same time. Ferroptosis may occur (1). As an oxidoreductase, NAD(P)H can be utilized by FSP1 to catalyze the regeneration of CoQ10 (CoQ10), inhibiting the reduced form of ubiquinone from capturing lipid peroxidation free radicals, thus depressing Ferroptosis (1, 28).

#### 3.3.2 The Mechanism of Promoting the Synthesis of Lipid Peroxides

Free long-chain polyunsaturated fatty acids (PUFAs) can be found in acyl-CoA synthase long-chain family 4 (ACSL4), oxygenases (LOXs) and lysophosphatidylcholine acyltransferase 3 (LPCAT3) in cells. Under the successive action of these enzymes, acylation and lipidation oxidation occurs to generate lipid peroxides. When its level exceeds the threshold, Ferroptosis occurs. Among the many lipid peroxides, AA-OOH-PE is the most representative and shows a strong correlation. Therefore, the accumulation of lipid peroxides mentioned above also mainly refers to the accumulation of AA-OOH-PE. In addition, long-chain polyunsaturated fatty acids are converted into lipid peroxides through the Fenton reaction and lipid auto-oxidation reaction (26).

However in fact, When in a normal physiological state, due to the presence of GPx4, the lipid peroxides produced by the above three pathways cannot accumulate in the body to induce Ferroptosis. Only in the case of glutathione deficiency, this oxidation of polyunsaturated fatty acids can cause great damage to cells. Therefore, we conclude that the loss, depletion or inactivation of glutathione may contribute more to Ferroptosis than the oxidation of polyunsaturated fatty acids; In other words, the process of inhibiting the reduction of lipid peroxides has a more significant effect on the initiation of Ferroptosis than the process of promoting the formation of lipid peroxides (24).

At the same time, studies have found that not all LOX inhibitors can save Ferroptosis, which suggests that lipid auto-oxidation may be the final process of Ferroptosis, rather than lipid peroxidation controlled by LOXS. Therefore, Shah R et al. put forward the hypothesis that lipid peroxides are relevant to the initiation of Ferroptosis. Once Ferroptosis begins, the lipid auto-oxidation reaction leads to the final cell death (29). The lipophilic free radical trapping antioxidants (RTAs), such as α-tocopherol, protect cells from auto-oxidation, thereby reducing the damage caused by Ferroptosis (24).

#### 3.3.3 The Effect of Iron Catalysis and Iron Homeostasis on Ferroptosis

Fe(II) and Fe(III), as a common endogenous catalyst, can increase the synthesis of lipid peroxides and accelerate the occurrence of Ferroptosis by promoting the Fenton reaction, phospholipid esterification oxidation reaction, and lipid autooxidation reaction. In addition to catalyzing the Fenton reaction to generate peroxygen free radicals and lipid peroxides, iron can also promote the electronic reaction of oxygen in mitochondria to generate superoxide and hydrogen peroxide (26). The existence of these four free radicals makes the occurrence of Ferroptosis accompanied by an inflammatory reaction and causes certain inflammatory damage (30).

When in a normal physiological state, the iron level in the body is usually in a dynamic equilibrium state. This iron homeostasis depends on iron regulatory protein (IRP1\IRP2), divalent metal transporter (DMT1), transferrin receptor 1 (TfR1), The combined effect of ferritin (FPN, composed of FTH1 and FTL) and transferrin (31). Transferrin (Tf) shows a high affinity for iron ions [III], and after the two are combined, they are transported into the cell matrix through the transferrin receptor 1 (TfR1) (30). At the same time, iron ions [III] can be transformed into ferrous ions under the effect of reductase. Enter the cell through a variety of transport systems, such as divalent metal transporter (DMT1) and type I voltage-dependent calcium channel (VDCC). Most of the iron in the matrix is taken up by mitochondria to synthesize heme and iron-sulfur clusters, and a few are reserved in ferritin (FPN). FPN can not only participate in the process of iron transfer to the outside of the cell, but also can be combined with nuclear receptor co-activator 4 (NCOA4) and transported to the lysosome to degrade and release iron, thereby increasing the iron content in the cell (25). Therefore, some studies have shown that NCOA4-mediated iron bacteriophages can promote Ferroptosis by increasing the intracellular iron content (22). It was also found that in cells sensitive to Ferroptosis, Tf increased and FPN decreased, which destroyed the iron homeostasis of the cell, raising local iron content, and induced Ferroptosis (23).

### 3.4 Detection of Ferroptosis

**Table:** 

Detection method of Ferroptosis	*in vitro*	Cell viability	calcei-nacetoxymethyl ester (Calcein AM) viability assay (23)
			trypan blue assay (23)
			Cell Counting Kit-8 assay
		Iron level	Fingelin cell membrane permeability dye-flow cytometry or confocal microscopy: monitoring the iron content in living cells (23)
		Ros level	C11-bodipy probe (23)
	*in vivo*	Prostaglandin-endoperoxide synthase (PTGS)	After the mice were treated with RSL3 and Erastin, PTGS2 encoding cox-2 was significantly up-regulated. Its rise marks the occurrence of Ferroptosis and does not affect its development (23).

### 3.5 The Relationship Between Ferroptosis and Disease Cancer

It has been found that some cancer cells can integrate the free long-chain polyunsaturated fatty acids PUFAS into the cell membrane, thereby showing a high dependence on glutathione peroxidase 4GPx4 (26). For example, triple-negative breast cancer is a kind of tumor that is difficult to treat, but recent studies have found that this type of cancer cell ACSL4 expresses and manifests PUFAS in the cell membrane, making it sensitive to Ferroptosis (32).

#### 3.5.1 Neurodegenerative Diseases

In recent years, people have gradually discovered that there are also characteristic phenomena of Ferroptosis in the development of neurodegenerative diseases, such as increased lipid peroxidation, decreased glutathione, and GPx4 inhibition. This phenomenon suggests that neurodegenerative diseases may be related to Ferroptosis (33). Iron gradually accumulates in the brain as we age. Iron accumulation is associated with the development of neurodegenerative diseases. Such as Alzheimer’s disease, which is common in old age, as well as Parkinson’s disease and AMyotrophic lateral sclerosis (34).

#### 3.5.2 Liver Damage

The impact of Ferroptosis on the liver is mainly drug-induced damage. For example, excessive intake of acetaminophen can be metabolized into acetophenone imine, which has the effect of depleting glutathione, which can cause Ferroptosis of liver cells and lead to liver damage (23). Some factors (for example, HBV, HCV infection, excessive alcohol intake, metabolic or genetic diseases, IR damage) can also trigger chronic liver disease or autoimmune liver disease ALD in the liver, causing the accumulation of iron, promoting the oxidation reaction, which is DNA and beneficial Protein damage and lipid peroxidation occurs. It will eventually induce Ferroptosis, gradually fibrosis and hardening of the liver, and even result in liver cancer (25).

#### 3.5.3 Kidney Damage

In 2019, Guerrero-Hue M’s experiment found for the first time that Ferroptosis is associated with rhabdomyolysis-mediated kidney damage. At the same time, it was found that both Ferroptosis inhibitors and curcumin inhibit the process, which can be regarded as a potential treatment for this symptom. For example, the third-generation iron statins (SRS16-86) inhibited Ferroptosis, limiting acute ischemia-reperfusion injury and acute renal failure AKF associated with oxalate nephropathy (23). Ferroptosis of tubular epithelial cells after ischemia-reperfusion injury will be inhibited by XJB-5-131 (35).

## 4 Ischemia-Reperfusion Injury And Ferroptosis

### 4.1 The First Discovery of the Relationship Between the Two

Although Ferroptosis was only officially defined in 2012 (21), the fact that iron is involved in the reperfusion injury process has been reported as early as the end of the 20th century (1). It was discovered in 1990 that iron chelating agents can reduce the generation of free radicals and prevent cardiomyocyte damage by eliminating the catalytic effect of iron in the Haber-Weiss redox reaction or inhibiting the iron-dependent lipid peroxidation reaction. So it is applied to the treatment of myocardial ischemia reperfusion injury (36). In the same year, it was also discovered that iron chelating agents have the effect of inhibiting the death of renal tubular cells and can be applied to the treatment of ischemia-reperfusion injury in the kidneys (37). The study of cerebral ischemia-reperfusion injury during the same period found that iron chelating agent has a mitigating effect on rodent models, but there is no noticeable effect in the human brain (1). In summary, in the mechanism of ischemia-reperfusion injury, Ferroptosis has its place very early.

### 4.2 Report on the Role of Ferroptosis in Ischemia-Reperfusion Injury in Various Tissues

In recent years, we have found many clues to prove that Ferroptosis plays an indelible role in the process of ischemia-reperfusion injury. Among them, there are many related studies on the brain, heart, and kidney. The main body of the determination process is similar, which can be summarized as the initial discovery that iron chelating agents can be used to reduce ischemia-reperfusion injury, and then it was discovered that blocking or promoting Ferroptosis-related processes can effectively reduce or aggravate ischemia-reperfusion injury. So far, it has been clear that a variety of Ferroptosis inhibitors can effectively alleviate and prevent the injury of ischemia-reperfusion injury. For example, ACSL4 can play an essential role in intestinal ischemia-reperfusion injury, and we can protect it by inhibiting ACSL4 and Ferroptosis inhibitors (1).

**Figure 3:**
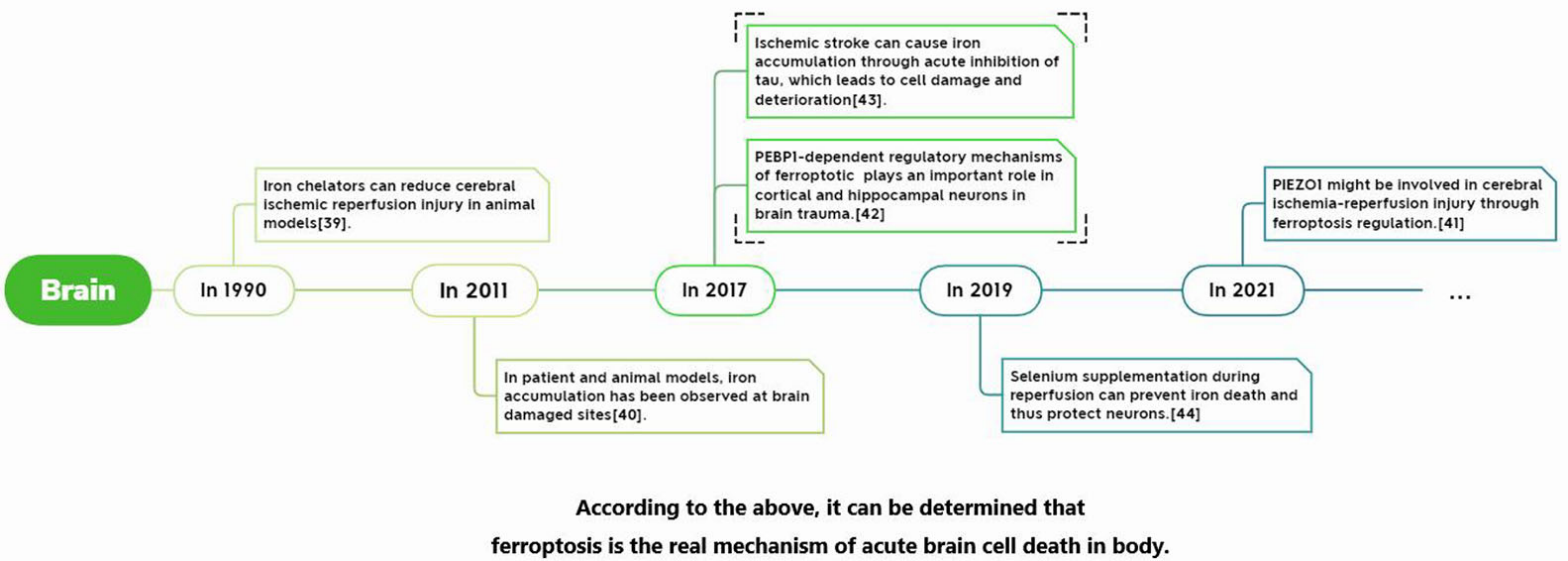
Report of Ferroptosis in cerebral ischemia-reperfusion injury.

**Figure 4:**
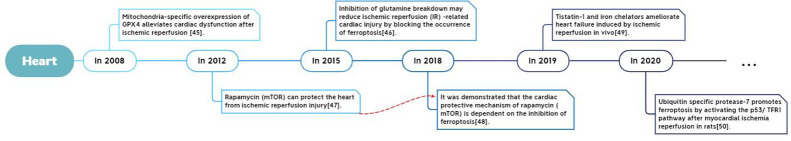
Report of Ferroptosis in cardiac ischemia-reperfusion injury.

**Figure 5:**
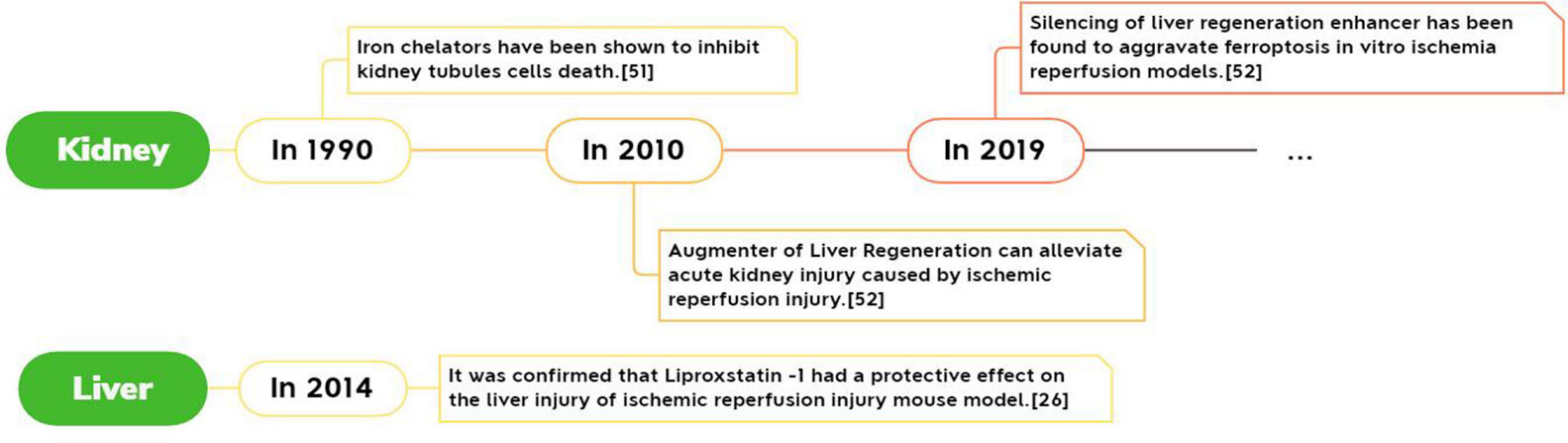
Report of Ferroptosis in liver and kidney ischemia-reperfusion injury.

Its development process in specific organs is shown in **Figure 3**–**5**.

### 4.3 The Feasibility Prospect of Ferroptosis as a Diagnostic Basis and treAtment Target for Ischemia-Reperfusion Injury

At present, more and more studies have found that the increase in lipid peroxidation and the increase in intracellular iron levels in ischemia-reperfusion injury are the same as the iron-dependent non-apoptotic form of Ferroptosis, and it has been confirmed that this damage can be prevented by iron chelating agents or antioxidants (38). The similarities that have been continuously discovered between the two have aroused widespread attention in the scientific community. In 2019, it was discovered that Ferroptosis was the first event in the initial stage of tissue damage, followed by other cell death pathways. Such as apoptosis, necrosis, etc. and secondary activation of necrotizing inflammation (39). In other words, Ferroptosis is the first step of ischemia-reperfusion injury, and the subsequent cell necrosis and inflammatory response are all subsequent steps secondary to Ferroptosis. This experiment once again proved this view (16). This point of view suggests that we need to shift the focus of the research and development of drugs for ischemia-reperfusion injury to inhibit the occurrence of Ferroptosis in the future.

#### 4.3.1 Therapeutic Targets Related to the Up-Regulation of SLC7A11

##### 4.3.1.1 Nrf2

Studies by Dong, H et al., have shown that Nrf2 can inhibit Ferroptosis by regulating SLC7A11 and HO-1, which may provide a potential strategy for the treatment of acute lung injury induced by intestinal ischemia/reperfusion (40). Similarly, studies have shown that pachymaric acid (PA) can directly or indirectly activate NRF2 or up-regulate the downstream Ferroptosis-related protein GPX4, XC system and heme oxygenase-1 (HO-1). To inhibit acute kidney injury (IRI-AKI) caused by ischemia-reperfusion caused by Ferroptosis (41).

##### 4.3.1.2 USP22

USP22, as one of the deubiquitinase family, is a new target can be used to treat cardiac reperfusion injury. It can inhibit the transcriptional expression of P53 by stabilizing the level of protein deacetylase sirtuin-1 (SIRT1), and increase the level of SLC7A11, thereby inhibiting Myocardial cell death induced by Ferroptosis in myocardial ischemia-reperfusion injury (42).

##### 4.3.1.3 Mir-182-5p and Mir-378a-3p

Recently, some researchers have also found *in vitro* experiments that miR-182-5p and miR-378a-3p can directly bind to the mRNA of GPX4 and SLC7A11 to negatively regulate GPX4 and the Expression of SLC7A11. And subsequent *in vivo* studies have indicated that silencing miR-182-5p and miR-378a-3p can alleviate IR-induced kidney damage in rats, which can be used as a potential therapeutic method for myocardial IR therapy (43).

#### 4.3.2 Therapeutic Targets Related to ACSL4 Downregulation

##### 4.3.2.1 Inhibition of Sp1

Special protein 1 (Sp1) can promote the transcriptional expression of ACSL4. Inhibition of ACSL4 before reperfusion can reduce the damage caused by Ferroptosis. This suggests that inhibition of Sp1 may become a unique and effective new way to prevent and treat intestinal IR injury (44).

#### 4.3.3 Targets Related to Stable Intracellular Iron Levels

##### 4.3.3.1 FtMt

Mitochondrial ferritin (FtMt) is an iron storage protein, which belongs to FTH (H-type ferritin). It is expressed in the cells of the central nervous system to the thymus, kidney, heart and many other organs. High levels of FtMt can reduce cytoplasmic iron content, reduce mitochondrial LIP and cytoplasmic LIP, resulting in an overall reduction in ROS production. Therefore, FtMt overexpression can inhibit cell death and ROS production. Therefore, it is inferred that FtMt may be a potential new targeting molecule for balancing iron homeostasis and reducing ROS therapy (31).

#### 4.3.4 Complementary Therapy

##### 4.3.4.1 Coenzyme Q10

Coenzyme Q10 is an endogenous fat-soluble antioxidant that can effectively inhibit lipid peroxidation and is expected to become a new Ferroptosis inhibitor. In 2019, oral coenzyme Q10 can significantly improve the prognosis of neurological damage in the rat MCAO model and patients with acute ischemic stroke (45).

##### 4.3.4.2 Selenium (Se)

Appropriate supplementation of selenium (Se) can promote the expression of GPX4, thereby effectively inhibiting GPX4-dependent Ferroptosis and cell death caused by endoplasmic reticulum stress (46).

#### 4.3.5 New Discoveries of Traditional Chinese Medicine Compound in Reducing IRI Ferroptosis Damage

Naotai Recipe is a compound Chinese medicinal preparation consisting of Astragalus and Chuanxiong as the main prescription, earthworm and silkworm to clear the meridian, relieve phlegm and eliminate wind. The occurrence of Ferroptosis can be inhibited by reducing the intracellular transfer of iron or enhancing the activity of GPx4. Traditional Chinese medicine still has a lot of research space in the direction of inhibiting Ferroptosis (47).

#### 4.3.6 Multi-target Combination Therapy Becomes a New Direction in the Future

In 2017, in young or old rat models, TUO et al. observed that knocking out the tubulin gene Tau can significantly reduce Ferroptosis and inhibit cerebral ischemia-reperfusion injury. But for old mice, the combined action of iron chelator and Tau gene knockout can reverse its cerebral ischemia-reperfusion injury (48). This implies that in the future, ischemia-reperfusion injury may require a combination of multiple treatments to achieve more effective control results.

### 4.4 Conclusion

With years of hard work by scientific researchers, it can be suggested that Ferroptosis is involved in ischemia-reperfusion in the body and plays an indelible role in the early stage. However, Ferroptosis inhibitors are rarely used in current clinical treatment. The result may be related to the fact that there are fewer Ferroptosis drugs on the market, and it may also be related to the fact that Ferroptosis inhibitors alone cannot effectively prevent the occurrence of reperfusion injury. In combination with the above, it can be seen that ischemia-reperfusion injury is caused by multiple pathways, and various pathways can also influence each other. All of the above remind us that ischemia-reperfusion injury requires a combination of multiple treatments to achieve more effective results. And future studies may be biased towards exploring the use of ferroptosis inhibitors in combination with other treatment options.

## Data Availability Statement

The original contributions presented in the study are included in the article/supplementary materials. Further inquiries can be directed to the corresponding author.

## Author Contributions

All authors listed have made a substantial, direct, and intellectual contribution to the work and approved it for publication.

## Conflict of Interest

The authors declare that the research was conducted in the absence of any commercial or financial relationships that could be construed as a potential conflict of interest.

## Publisher’s Note

All claims expressed in this article are solely those of the authors and do not necessarily represent those of their affiliated organizations, or those of the publisher, the editors and the reviewers. Any product that may be evaluated in this article, or claim that may be made by its manufacturer, is not guaranteed or endorsed by the publisher.
